# Syphilis in the Middle Ages and in Modern Times

**Published:** 1896-06

**Authors:** 


Syphilis in the Middle Ages and in Modern Times. By Dr. F. Buret, Paris, France. Translated from the French, with notes, by A. H. Ohmann-Dumesnil, M. D., Professor of Dermatology and Syphilology in the Marion Sims College of Medicine; Consulting Dermatologist to the St. Louis City Hospital, to the St. Louis Female Hospital; Physician for

Cutaneous Diseases to the Alexian Brothers' Hospital; Dermatologist to Pius Hospital, to the Rebekah Hospital, to the St. Louis Polyclinic and Emergency Hospital, etc., etc. Being Volumes II and III, of *Syphilis To-Day and Among the Ancients*, complete in three volumes. 12mo. 300 pages. Extra Cloth, $1.50 net. Philadelphia: The F. A. Davis Co., Publishers, 1914 and 1916 Cherry Street.

The first volume of this remarkable book has been noticed before. In The Homœopathic Physician for September, 1892, at page 409, is a review in which the salient features of the book have been faintly outlined.

The second and third volumes of that work are now before us, bound as one volume.

If the first volume was remarkable in its revelations concerning the antiquity of syphilis, these two later volumes are still more remarkable. The proofs given of the presence of syphilis before the discovery of America and before the siege of Naples are amazing. They show immense and patient research and a fine instinct in selecting the right paths in which to find the desired testimony to prove the stand taken by the author.

The three volumes taken together form a very vivid history of the early phases of syphilis.

In ancient times, and in the middle ages, the nature of syphilis was not understood, and though certain phenomena and symptoms were known to arise by contact with lewd women, the full significance of these phenomena was not imagined. Constantly, through all these long periods of time, the disease was confounded with leprosy. Indeed the most obvious cases of syphilis were called leprosy.

In consequence of this error there arose a great variety of opinions upon the contagiousness of leprosy. This conflict of opinion continues to this day. Dr. Zambaco, of Constantinople, in a communication to the Paris Academy, on August 13th, 1889, declared that while leprosy is hereditary it is not contagious. Professor Leloir, of Lille, on the other hand, declares that it is contagious; and he is again offset by Dr. Springer, who after studying the lepers of Norway, casts a doubt upon the contagiousness of the disease.

Meanwhile the discovery of the bacillus of leprosy has once more "restored the doctrine of contagion."

In the face of this restoration, Professor Cornil once more throws doubt on the doctrine of contagion.

This state of confusion in which it is involved would seem to render the problem hopeless. Nevertheless our author offers a solution. He asks: "Why could it not be regarded as a constitutional parasitic disease, analogous to tuberculosis, whose direct transmissibility would only take place under exceptional circumstances and in an already well-prepared soil?" Quoting Dr. Le Grand, he comes to the conclusion that "*there never was more*

*of an epidemic of leprosy than there has been an epidemic of syphilis or of phthisis.*"

Turning now to the consideration of the testimony concerning the character of leprosy and its symptoms, the author finds that this testimony reveals little else than a veiled history of syphilis.

Syphilis as a distinct disease, with a variety of manifestations, was not known. Leprosy was known by tradition at least. So when the phenomena of syphilis from contact with infected women appeared they were at once attributed to leprosy.

Instances are given where this mistake was obviously made. Thus, Gordon, a writer of the fourteenth century, says: "A certain countess who had leprosy went to Montpellier, and I treated her toward the end. A bachelor of medicine whom I had placed by her, shared her bed, and made her big with child; but he became *leprous* himself."

Another author relates the story of "a carpenter who, having had to do with a leprous woman, was infected with *leprosy* a short time after."

In contrast with these instances we have the statement of the modern Dr. Zambaco, before quoted, in reference to true leprosy, "that married men and women, perfectly healthy, may live with their consorts, who are notoriously leprous, without ever contracting the slightest trouble." Thus the author is "forced to the conclusion once more, that the word *leprosy* in the middle ages was most often the synonym of *venereal disease*."

Moreover, the author finds that there were authors preceding himself who held this opinion. One of these, Vercelloni, who wrote in Latin verse in 1701, says: "When I read the books of the ancients on Leprosy, it seems to me that I am reading works on the pox, and the titles alone of these books persuade me that they are treatises on this disease."

A cemetery connected with a leper asylum of the middle ages, in Paris, was dug up a few years ago and the bones critically examined. They were profoundly altered, and all in the same manner. Yet these lesions did not answer at all with those of leprosy, while they did resemble the ravages of syphilis.

It is not to our taste, nor is it needful, to go into all the nauseous details and translate all the hints of human depravity to be found in this volume, valuable as they all are in establishing the truth concerning the antiquity of syphilis.

Suffice it to quote from the preface that the proofs are irrefutable "that the appearance of syphilis is lost in the twilight of time, and that the disease has no age. It is found described, in fact, among the most ancient peoples—even among the Assyrians and Babylonians—under pretty similar forms, by priests and poets rather than by physicians." "But," adds the author with characteristic sarcasm, "it must be admitted that the physicians, in these remote times, did not seem to enjoy the confidence which is accorded them to-day."

